# Integrative Therapy Combining Chinese Herbal Medicines With Conventional Treatment Reduces the Risk of Cardiovascular Disease Among Patients With Systemic Lupus Erythematosus: A Retrospective Population-Based Cohort Study

**DOI:** 10.3389/fphar.2021.737105

**Published:** 2021-09-27

**Authors:** Han-Hua Yu, Chia-Jung Hsieh

**Affiliations:** ^1^ Division of Rheumatology, Allergy and Immunology, Linkou Chang Gung Memorial Hospital, Taoyuan, Taiwan; ^2^ Department of Chinese Medicine, Hualien Tzu Chi Hospital, Buddhist Tzu Chi Medical Foundation, Hualien, Taiwan; ^3^ Department of Public Health, Tzu Chi University, Hualien, Taiwan

**Keywords:** systemic lupus erythematosus (SLE), Chinese medicine, cardiovascular disease, herb, cohort

## Abstract

Systemic lupus erythematosus (SLE) is a chronic systemic autoimmune disease that involves multiple systems and organs. Advanced conventional treatment does not appear to markedly reduce the risk of cardiovascular disease (CVD) among patients with SLE. Chinese medicine is a complementary and alternative medicine system, and some SLE patients in Taiwan also use Chinese herbal medicines (CHMs). Thus, we aimed to investigate whether integrative therapy combining CHMs with conventional therapy reduces the risk of CVD among patients with SLE. We performed a 12-years population-based retrospective cohort study using the “Systemic Lupus Erythematosus Health Database” of the National Health Insurance Research Database (NHIRD) in Taiwan. Patients newly diagnosed with SLE between 2004 and 2013 were divided into CHM and non-CHM groups and followed up until the end of 2015. We applied 1:1 individual matching by age, gender, and year of being newly diagnosed with SLE; accordingly, 2,751 patients were included in both CHM and non-CHM groups after matching. We applied the Cox proportional hazard regression model to determine the risk of CVD in relation to CHM use. During the follow-up period, 407 patients in the CHM group and 469 patients in the non-CHM group developed CVD, with incidence rates of 337 and 422 per 10,000 person-years, respectively. The Cox proportional hazards model demonstrated a significantly decreased risk of CVD among SLE patients using CHMs (adjusted HR: 0.83; 95% CI 0.73–0.95; *p* = 0.008). Further analyses of different types of CVDs also showed a significantly decreased risk of ischemic stroke in the CHM group (adjusted HR: 0.74; 95% CI 0.57–0.97; *p* = 0.032). Among the frequently used single herbs and polyherbal formulas, Shu-Jing-Huo-Xue-Tang was associated with a significantly decreased risk of CVD (adjusted HR: 0.76; 95% CI 0.58–0.99; *p* = 0.041). In conclusion, CHM use reduced the risk of CVD among patients with SLE in Taiwan. Further randomized studies may be needed to determine the definite causal relationship between CHM use and its protective effects against CVD among patients with SLE.

## Introduction

Systemic lupus erythematosus (SLE) is a chronic systemic autoimmune disease involving multiple systems and organs, and it presents with an unacceptably high morbidity burden and various complications (Golder and Hoi, 2017; Dörner and Furie, 2019; Durcan et al., 2019). Survival of SLE patients has improved over the past several decades. An international cohort study revealed a dramatic 60% decrease in the standardized mortality rate (SMR) among SLE patients (SMR in 1970–1979, 4.9; SMR in 1990–2001, 2.0) (Bernatsky et al., 2006). The 10-years survival rate now exceeds 90%, while the 20-years survival rate has increased to approximately 80% (Doria et al., 2006; Kasitanon et al., 2006; Durcan et al., 2019). This decrease in mortality may be attributable to early diagnosis and advanced treatment (Fors Nieves and Izmirly, 2016). However, the survival of patients with SLE has not continued to increase throughout the 2000s (Tektonidou et al., 2017).

Cardiovascular disease (CVD) is one of the most common causes of death among patients with SLE (Yee et al., 2015; Tektonidou et al., 2017). In assessments based on the specific causes of death, the SMRs due to infections and renal disorders showed a dramatic decrease, but the SMR due to circulatory diseases increased slightly from 1970 to 2001 (Bernatsky et al., 2006). A Taiwan National Health Insurance Research Database (NHIRD) cohort study demonstrated a 1.67-fold (95% CI 1.45–1.91) higher risk of ischemic stroke among patients with SLE than in the general population (Chiu et al., 2012). Another study showed a 5.11-fold (95% CI 2.63–9.92) higher risk of acute myocardial infarction among patients with SLE (Lin et al., 2014). A meta-analysis of population-based cohort studies revealed both significantly higher risk ratios of any stroke (risk ratio 2.53, 95% CI 1.96–3.26) and ischemic stroke (risk ratio 2.10, 95% CI 1.68–2.62) among patients with SLE than in the general population (Holmqvist et al., 2015). A Swedish population-based SLE cohort study that followed up patients during 1964–1995 also revealed that the hazard ratio (HR) of CVD in the latter 10-year period (1985–1994) did not decrease significantly from that in the earlier 10-year period (1964–1974) (Bjornadal et al., 2004). The possible mechanisms of CVD development among SLE patients include premature atherosclerosis, defective endothelial cell function and vascular repair, dysfunctional immune regulation, high density lipoprotein (HDL) dysfunction, increased oxidative stress, elevated homocysteine levels, and elevated leptin levels (Roman et al., 2003; Skaggs et al., 2012; Liu and Kaplan, 2018).

A questionnaire survey in Taiwan revealed that 46% of the patients with SLE also received complementary and alternative therapies (Chou, 2010). Traditional Chinese medicine has been clinically practiced for more than 2000 years and is one of the major complementary and alternative medicine systems worldwide (Liao et al., 2011). Although some studies have reported beneficial effects of Chinese herbal medicines (CHMs) among SLE patients, including improvement of symptoms (You et al., 2010), reduced disease activity (Liao et al., 2011; Zhong et al., 2013), fewer adverse effects of conventional drugs (Huang et al., 2013), reduced mortality (Ma et al., 2016), and a lower risk of lupus nephritis (Chang et al., 2017), there is still a lack of large-scale studies on the effects of CHMs on CVD among SLE patients.

Therefore, in this study, we used data from the NHIRD in Taiwan to perform a nationwide population-based retrospective cohort study and investigated whether integrative therapy combining CHMs with conventional therapy reduces the risk of CVD among SLE patients, in addition to analyzing the effects of different single herbs and polyherbal formulas of CHMs on CVD.

## Materials and Methods

### Data Source

The NHIRD in Taiwan was employed in this nationwide population-based retrospective cohort study. We used the “Systemic Lupus Erythematosus Health Database” from the Health and Welfare Data Science Center (HWDC) in Taiwan. This database defined a new diagnosis of SLE as more than three western medicine outpatient visits for code 710.0 of the International Classification of Disease, Ninth Revision, Clinical Modification (ICD-9-CM) per year with an interval of more than 4 weeks between visits. This study was approved by the Research Ethics Committee of Hualien Tzu Chi Hospital, Buddhist Tzu Chi Medical Foundation (IRB107-228-C).

### Study Population and Design

We initially included 19,811 patients newly diagnosed with SLE between 2004 and 2013. A total of 7,392 patients were excluded because they were not diagnosed with SLE by a rheumatologist, had missing gender or age data, were diagnosed with SLE at <20 years of age, or had CVD before SLE diagnosis. The remaining 12,419 patients were divided into CHM and non-CHM groups. Since some patients visited Chinese medicine outpatient departments for acupuncture, moxibustion, or manipulative therapy, we defined CHM group patients as those who visited Chinese medicine outpatient departments with CHM prescriptions. Since the effect of CHMs on the endothelium persists for at least 2 months (Chang et al., 2016), we defined patients in the non-CHM group by the absence of Chinese medicine outpatient department visits during the period from 2 months before SLE diagnosis until end of the study.

**FIGURE 1:**
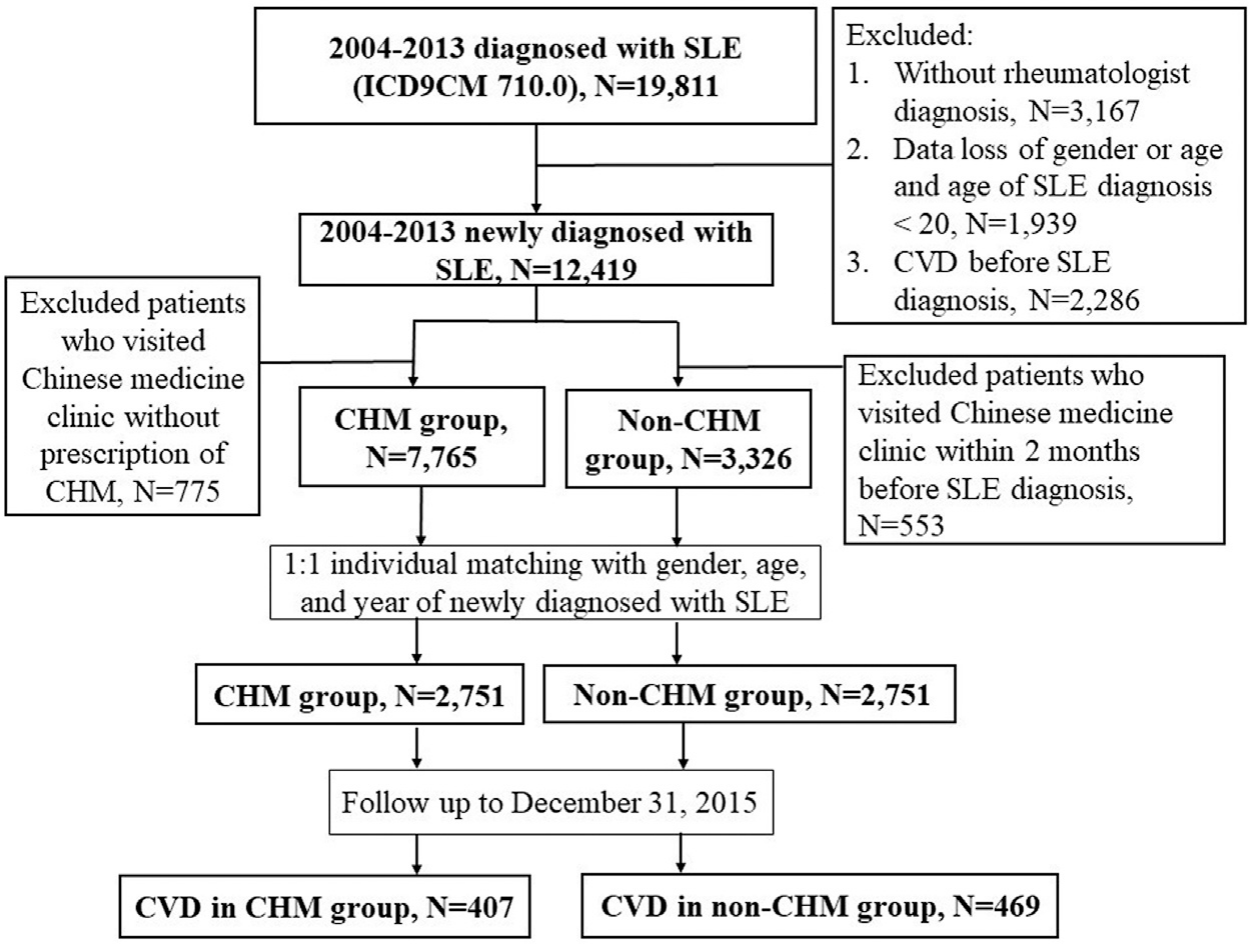
Flowchart of the study. SLE, systemic lupus erythematosus; ICD-9-CM, International Classification of Disease, Ninth Revision, Clinical Modification; CVD, cardiovascular disease; CHM, Chinese herbal medicine.

To avoid immortal time bias, we defined the same immortal time for both the CHM and non-CHM groups, i.e., the duration from the SLE diagnosis date to the index date. The index date of the CHM group was the first prescription for CHMs after SLE diagnosis. We applied 1:1 individual matching with gender, age, and year of newly diagnosed with SLE in both groups, and the index date of the non-CHM group was the date of newly diagnosed with SLE plus the immortal time of the matched CHM group. Both the CHM and non-CHM groups excluded patients showing CVD events prior to the index date. A total of 2,751 patients with SLE were finally included in both the CHM and non-CHM groups. All eligible patients were followed up from the index date until December 31, 2015, the initial diagnosis date of CVD, or until the date of death, whichever occurred first. Figure 1 shows the flowchart of the study.

### Study Variables

The primary outcome was CVD events during the 12-years follow-up period, which was defined as at least two outpatient department visits or at least one hospitalization. CVD was defined as atherosclerosis-associated disease based on the World Health Organization (WHO) criteria, and included coronary artery disease (CAD), ischemic stroke, and peripheral artery disease (PAD) (Mendis et al., 2011). The ICD-9-CM definitions of CVD were based on the codes used in previous studies, including 410–414 for CAD (Emdin et al., 2015), 433–436 for ischemic stroke (Barbhaiya et al., 2018), and 440.0/440.2/440.3/440.8/440.9/443/444.0/444.22/444.8/444.9/447.8/447.9 for PAD (Chang et al., 2013).

The urbanization level of patients’ insurance location was categorized according to a previous study of development stratification in Taiwanese townships by Liu et al. (2006). We defined the first cluster as high urbanization level, the second cluster as moderate urbanization level, and the third to the seventh clusters as low urbanization level.

Comorbidities and baseline drugs used in both the CHM and non-CHM groups were analyzed. Comorbidities were defined as at least two outpatient visits or at least one hospitalization before the index date. Comorbidities included hypertension (ICD-9-CM: 401), diabetes mellitus (ICD-9-CM: 250), dyslipidemia (ICD-9-CM: 272), end-stage renal disease (ICD-9-CM: 585), and atrial fibrillation (ICD-9-CM: 427.31). We defined the baseline drugs according to the history of drug use 1 year prior to the index date. Baseline drugs included nonsteroidal anti-inflammatory drugs (NSAIDs), corticosteroids, antiplatelet agents, anticoagulants, hydroxychloroquine, sulfasalazine, azathioprine, methotrexate, leflunomide, mycophenolic acid, cyclosporine, and cyclophosphamide. The drugs were classified according to the Anatomical Therapeutic Chemical (ATC) codes maintained by the WHO (https://www.whocc.no/atc_ddd_index).

### Statistical Analysis

Continuous data (including age, follow-up period and interval from SLE diagnosis date to index date) were presented as medians and interquartile ranges (IQRs) due to their non-normal distribution, and the Mann-Whitney U test was used for group comparisons. Categorical data (including gender, age classification, urbanization level, classification of insured amount, death, classification of interval from SLE diagnosis date to index date, comorbidity, and baseline drug) were presented as numbers and percentages, and the chi-squared test was used for group comparisons. In addition, to demonstrate the effect size, a standardized difference was also used for group comparisons (Cummings, 2011).

To analyze the CVD risk between the CHM and non-CHM groups, we used the Cox proportional hazards model with adjustment for factors, including age, gender, insured amount, urbanization level, comorbidities, and baseline drugs. Kaplan-Meier and log-rank test were used for group comparisons of cumulative incidence. Further analyses of different types of CVD (including CAD, ischemic stroke, and PAD) were also performed. To investigate the effect of CHMs on CVD in different subgroups, we performed stratification analysis of CVD risk in the CHM group in comparison with the non-CHM group. Moreover, the CVD risk associated with the top 10 frequently used single herbs/polyherbal formulas compared with that in the matched non-CHM group was analyzed.

Since the effect of CHMs on the endothelium takes time, sensitivity tests for different intervals between the index date and the initial diagnosis date of CVD were performed (Supplementary Material S1). Sensitivity tests for different intervals between the SLE diagnosis date and the index date were also conducted (Supplementary Material S2). In addition, a sensitivity test for different cumulative days of CHM use was also conducted (Supplementary Material S3). Serial sensitivity analyses are described in the supplementary materials.

A two-sided *p*-value of less than 0.05 was considered statistically significant in this study. Statistical analyses were performed using SAS 9.4 (SAS Institute Inc., Cary, NC, United States).

## Results

### Basic Characteristic

Table 1 shows the characteristics of SLE patients who received treatment with and without CHMs, including gender, age, urbanization level, insured amount, follow-up period, death, interval from SLE diagnosis date to index date, comorbidity, and baseline drug. After individual matching, the gender, age, and interval from the SLE diagnosis date to the index date were similar between the CHM and non-CHM groups. The CHM and non-CHM groups showed significant differences in the classification of the insured amount (29.7% of NT$0–22,799, 42.5% of NT$22,800–38,199, 27.8% of ≥NT$38,200 vs. 33.4% of NT$0–22,799, 41.4% of NT$22,800–38,199, 25.2% of ≥NT$38,200, *p* = 0.007), follow-up period (3.81 vs. 3.44, *p* < 0.001), mortality (6.0 vs. 9.5%, *p* < 0.001), hypertension (12.1 vs. 15.2%, *p* = 0.001), diabetes mellitus (4.3 vs. 5.8%, *p* = 0.012), dyslipidemia (7.0 vs. 9.4%, *p* = 0.001), end-stage renal disease (2.1 vs. 4.1%, *p* < 0.001), and the use of NSAIDs (83.4 vs. 77.6%, *p* < 0.001), anti-platelet agents (16.0 vs. 19.1%, *p* = 0.003), and disease-modifying antirheumatic drugs (DMARDs, including mycophenolic acid, cyclosporine, and cyclophosphamide, 7.3 vs. 9.2%, *p* = 0.009).

**TABLE 1:** Characteristics of systemic lupus erythematosus patients treated with and without Chinese herbal medicines.

	CHM group (*n* = 2,751)	Non-CHM group (*n* = 2,751)	*p*-value^a^	Standardized difference^b^
Gender				
Female	2,293 (83.4%)	2,293 (83.4%)	1.000	0
Male	458 (16.7%)	458 (16.7%)	—	0
Age (years)				
Median (IQR)	42 (31,53)	42 (31,54)	0.451	0.044
20–29	563 (20.5%)	563 (20.5%)	1.000	0
30–39	678 (24.7%)	678 (24.7%)	—	0
≥40	1,510 (54.9%)	1,510 (54.9%)	—	0
Urbanization level^c^				
Low	1,005 (36.6%)	1,066 (38.8%)	0.087	0.045
Moderate	958 (34.9%)	884 (32.2%)	—	0.057
High	786 (28.6%)	800 (29.1%)	—	0.011
Insured amount (NT$/month)^d^				
0–22,799	816 (29.7%)	918 (33.4%)	0.007	0.080
22,800–38,199	1,168 (42.5%)	1,139 (41.4%)	—	0.022
≥38,200	765 (27.8%)	693 (25.2%)	—	0.060
Follow-up period (year), median (IQR)	3.81 (2.30,6.23)	3.44 (1.95,5.74)	<0.001	0.125
Death	164 (6.0%)	262 (9.5%)	<0.001	0.134
Interval from SLE diagnosis date to index date (year)				
Median (IQR)	0.56 (0.13, 1.69)	0.56 (0.13, 1.69)	1.000	0
0–0.5	1,295 (47.1%)	1,295 (47.1%)	1.000	0
0.5–1	417 (15.2%)	417 (15.2%)	—	0
1–3	731 (26.6%)	731 (26.6%)	—	0
3–6	246 (8.9%)	246 (8.9%)	—	0
6–12	62 (2.3%)	62 (2.3%)	—	0
Comorbidities				
Hypertension	333 (12.1%)	417 (15.2%)	0.001	0.089
Diabetes mellitus	118 (4.3%)	159 (5.8%)	0.012	0.068
Dyslipidemia	193 (7.0%)	258 (9.4%)	0.001	0.086
ESRD	59 (2.1%)	112 (4.1%)	<0.001	0.111
Atrial fibrillation	8 (0.3%)	17 (0.6%)	0.071	0.049
Baseline drugs				
NSAIDs	2,295 (83.4%)	2,135 (77.6%)	<0.001	0.147
Corticosteroids	2,104 (76.5%)	2,104 (76.5%)	1.000	0
Antiplatelet agents	440 (16.0%)	524 (19.1%)	0.003	0.081
Anticoagulants	54 (2.0%)	64 (2.3%)	0.352	0.026
Hydroxychloroquine	1989 (72.3%)	1988 (72.3%)	0.976	0.001
Sulfasalazine	166 (6.0%)	194 (7.1%)	0.127	0.041
DMARDs^e^	690 (25.1%)	744 (27.0%)	0.097	0.045
DMARDs^f^	200 (7.3%)	253 (9.2%)	0.009	0.070

a Mann-Whitney U test for continuous data and chi-squared test for categorical data.

b Calculation of effect size: mean for continuous data and percentage for categorical data.

c Number of data loss: 3.

d Number of data loss: 3.

e Azathioprine, methotrexate, and leflunomide.

f Mycophenolic acid, cyclosporine, and cyclophosphamide.

CHM, Chinese herbal medicine; ESRD, end-stage renal disease; NSAIDs, nonsteroidal anti-inflammatory drugs.

In addition, when we compared the CHM and non-CHM groups on the basis of standardized differences, the effect sizes of all characteristics were less than 0.2. Thus, according to previous studies on standardized differences (Cummings, 2011; Wu et al., 2018), the differences in all characteristics between the groups in the present study were negligible.

### Incidence and Risk of Cardiovascular Disease

**FIGURE 2:**
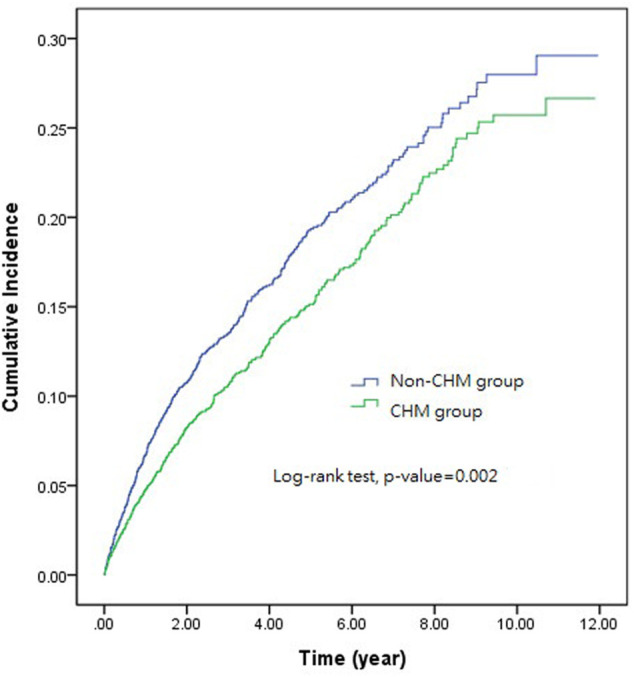
The cumulative incidence of cardiovascular disease between systemic lupus erythematosus patients treated with and without Chinese herbal medicines.CHM, Chinese herbal medicine.

At the end of the study, 407 SLE patients using CHMs developed CVD, while 469 SLE patients who were not using CHMs developed CVD. Table 2 demonstrates the incidence rates and incidence rate ratios of CVD classified according to CHM use. The incidence rate of CVD was significantly lower in the CHM group than in the non-CHM group. Further analyses of different types of CVDs (including CAD and ischemic stroke) also revealed significantly lower incidence rates in the CHM group (Table 2). Comparison of the cumulative incidence of CVD between the CHM and non-CHM groups was performed using Kaplan-Meier and log-rank test (Figure 2). The cumulative incidence of CVD significantly decreased among SLE patients using CHMs (log-rank test, *p* = 0.002).

**TABLE 2:** Incidence rates and incidence rate ratios of cardiovascular disease in systemic lupus erythematosus patients treated with and without Chinese herbal medicines.

	Patients	Person-years	Event	IR (95%CI) per 10,000 person-years	IRR (95%CI)
CVD					
Non-CHM group	2,751	11102.3	469	422.4 (385.9–462.4)	1.0
CHM group	2,751	12077.7	407	337.0 (305.8–371.4)	0.80 (0.70–0.91)*
CAD					
Non-CHM group	2,751	11168.7	199	178.2 (155.1–204.7)	1.0
CHM group	2,751	12114.0	168	138.7 (119.2–161.3)	0.78 (0.63–0.96)*
Ischemic stroke					
Non-CHM group	2,751	11172.4	125	111.9 (93.9–133.3)	1.0
CHM group	2,751	12127.2	95	78.3 (64.1–95.8)	0.70 (0.54–0.91)*
PAD					
Non-CHM group	2,751	11137.5	216	193.9 (169.7–221.6)	1.0
CHM group	2,751	12105.3	194	160.3 (139.2–184.5)	0.83 (0.68–1.00)

Model 1: adjusted for age, gender, insured amount, and urbanization level.

Model 2: adjusted for age, gender, insured amount, urbanization level, comorbidities, and baseline drugs.

IR, incidence rate; IRR, incidence rate ratio; CVD, cardiovascular disease; CHM, Chinese herbal medicine; CAD, coronary artery disease; PAD, peripheral artery disease.

*p-value < 0.05.

Table 3 shows the findings of the univariate and multivariate Cox proportional hazard models used for the cohort of SLE patients with CVD. Multivariate analysis demonstrated a significant decrease in the CVD risk among SLE patients using CHMs (adjusted HR: 0.83; 95% CI 0.73–0.95; *p* = 0.008). Further analyses of different types of CVD (including CAD, ischemic stroke, and PAD) also demonstrated a significant decrease in the ischemic stroke risk among the SLE patients using CHMs (adjusted HR: 0.74; 95% CI 0.57–0.97; *p* = 0.032).

**TABLE 3:** Hazard ratios of cardiovascular disease in systemic lupus erythematosus patients treated with and without Chinese herbal medicines.

	Crude HR (95% CI)	Adjusted HR (95% CI)	
		Model 1	Model 2
CVD			
Non-CHM group	1.0	1.0	1.0
CHM group	0.81 (0.71–0.93)*	0.82 (0.71–0.93)*	0.83 (0.73–0.95)*
CAD			
Non-CHM group	1.0	1.0	1.0
CHM group	0.79 (0.64–0.97)*	0.79 (0.64–0.97)*	0.82 (0.67–1.01)
Ischemic stroke			
Non-CHM group	1.0	1.0	1.0
CHM group	0.70 (0.53–0.91)*	0.71 (0.54–0.92)*	0.74 (0.57–0.97)*
PAD			
Non-CHM group	1.0	1.0	1.0
CHM group	0.85 (0.70–1.03)	0.85 (0.70–1.03)	0.85 (0.70–1.03)

Model 1: adjusted for age, gender, insured amount, and urbanization level.

Model 2: adjusted for age, gender, insured amount, urbanization level, comorbidities, and baseline drugs.

HR, hazard ratio; CI, confidence interval; CVD, cardiovascular disease; CHM, Chinese herbal medicine; CAD, coronary artery disease; PAD, peripheral artery disease.

*p-value < 0.05.

### Stratification Analysis

**FIGURE 3:**
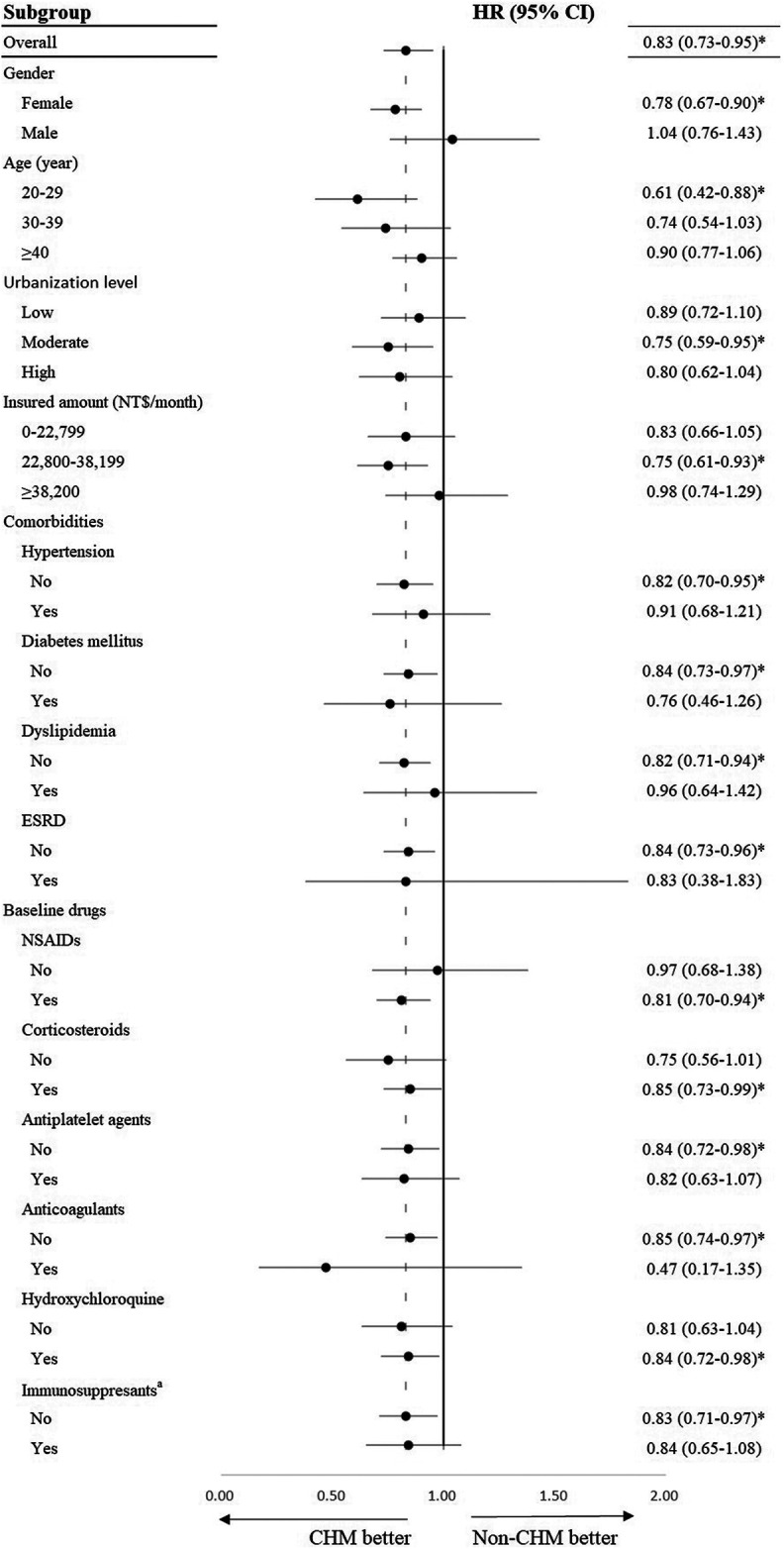
Stratification analysis of hazard ratios of cardiovascular disease among systemic lupus erythematosus patients treated with Chinese herbal medicines (in comparison with systemic lupus erythematosus patients treated without Chinese herbal medicine). Adjusted for age, gender, insured amount, urbanization level, comorbidities, and baseline drugs. HR, hazard ratio; CI: confidence interval; ESRD, end-stage renal disease; NSAID, nonsteroidal anti-inflammatory drug; CHM, Chinese herbal medicine. ^a^ Azathioprine, methotrexate, leflunomide, mycophenolic acid, cyclosporine, and cyclophosphamide. *p-value < 0.05.

Figure 3 demonstrates the HR of CVD among CHM users in comparison with non-CHM users in different subgroups. The following subgroups of CHM users showed significantly lower CVD risk: females, patients aged 20–29 years, those living in moderate urbanization level, those with an insured amount of NT$22800–38199/month, patients without hypertension, patients without diabetes mellitus, patients without dyslipidemia, patients without end-stage renal disease, NSAIDs users, corticosteroids users, hydroxychloroquine users, non-antiplatelet agent users, and non-anticoagulant users.

### Frequently Used Single Herbs and Polyherbal Formulas

Table 4 shows the HR of CVD in relation to the top 10 frequently used single herbs compared with that in the matched non-CHM groups. The most frequently used single herb was Corydalis yanhusuo (Y.H.Chou and Chun C.Hsu) W.T.Wang ex Z.Y.Su and C.Y.Wu (Yan Hu Su; 786 users, 28.6%). Patients using Rehmannia glutinosa (Gaertn.) DC. (Sheng Di Huang) showed a significant decrease in the HR of CVD (adjusted model 1: 0.73; 95% CI 0.54–0.98; *p* = 0.037). Table 5 shows the HR of CVD for the top 10 frequently used polyherbal formulas in comparison with the matched non-CHM groups. The most frequently used polyherbal formula was Jia-Wei-Xiao-Yao-San (975 users, 35.4%). Patients using Shu-Jing-Huo-Xue-Tang showed a significant decrease in the HR for CVD (adjusted HR: 0.76; 95% CI 0.58–0.99; *p* = 0.041).

**TABLE 4:** Hazard ratios of cardiovascular disease for the top 10 frequently used single herbs in comparison with the matched non-CHM groups.

Single herbs	Patients/total CHM users (%)	Adjusted HR (95% CI)	
		Model 1	Model 2
Corydalis yanhusuo (Y.H.Chou & Chun C.Hsu) W.T.Wang ex Z.Y.Su & C.Y.Wu (Yan Hu Suo)	786/2,751 (28.6%)	0.81 (0.64–1.02)	0.81 (0.64–1.02)
Salvia miltiorrhiza Bunge (Dan Shen)	598/2,751 (21.7%)	1.02 (0.79–1.33)	1.01 (0.77–1.32)
Rehmannia glutinosa (Gaertn.) DC. (Sheng Di Huang)	573/2,751 (20.8%)	0.73 (0.54–0.98)*	0.77 (0.57–1.05)
Astragalus mongholicus Bunge (Huang Qi)	565/2,751 (20.5%)	0.98 (0.75–1.29)	0.99 (0.75–1.31)
Scrophularia ningpoensis Hemsl. (Xuan Shen)	557/2,751 (20.3%)	0.83 (0.63–1.09)	0.86 (0.65–1.14)
Paeonia × suffruticosa Andrews (Mu Dan Pi)	548/2,751 (19.9%)	0.87 (0.65–1.16)	0.89 (0.67–1.19)
Pueraria montana var. lobata (Willd.) Maesen & S.M.Almeida ex Sanjappa & Predeep (Ge Gen)	544/2,751 (19.8%)	0.87 (0.67–1.14)	0.88 (0.67–1.16)
Platycodon grandiflorus (Jacq.) A.DC. (Jie Geng)	536/2,751 (19.5%)	0.86 (0.65–1.13)	0.88 (0.67–1.17)
Aconitum carmichaeli Debeaux (Fu Zi)	532/2,751 (19.3%)	0.87 (0.67–1.14)	0.89 (0.67–1.16)
Rheum palmatum L. (Da Huang)	531/2,751 (19.3%)	0.82 (0.62–1.08)	0.86 (0.65–1.15)

Model 1: adjusted for age, gender, insured amount, and urbanization level.

Model 2: adjusted for age, gender, insured amount, urbanization level, comorbidities, and baseline drugs.

HR, hazard ratio; CHM, Chinese herbal medicine; CI, confidence interval.

*p-value < 0.05.

**TABLE 5:** Hazard ratios of cardiovascular disease for the top 10 frequently used polyherbal formulas in comparison with the matched non-CHM groups.

Polyherbal formulas	Patients/total CHM users (%)	Adjusted HR (95%CI)	
		Model 1	Model 2
Jia-Wei-Xiao-Yao-San	975/2,751 (35.4%)	0.87 (0.70–1.08)	0.91 (0.73–1.13)
Shao-Yao-Gan-Cao-Tang	636/2,751 (23.1%)	0.82 (0.63–1.05)	0.85 (0.66–1.10)
Ge-Gen-Tang	618/2,751 (22.5%)	0.77 (0.59–1.01)	0.80 (0.61–1.05)
Xiao-Chai-Hu-Tang	588/2,751 (21.4%)	0.79 (0.61–1.03)	0.82 (0.63–1.08)
Shu-Jing-Huo-Xue-Tang	585/2,751 (21.3%)	0.74 (0.57–0.96)*	0.76 (0.58–0.99)*
Zhi-Bo-Di-Huang-Wan	541/2,751 (19.7%)	0.83 (0.62–1.10)	0.89 (0.66–1.18)
Liu-Wei-Di-Huang-Wan	533/2,751 (19.4%)	0.79 (0.60–1.05)	0.83 (0.62–1.10)
Gan-Lu-Yin	529/2,751 (19.2%)	0.88 (0.67–1.16)	0.95 (0.72–1.25)
Ban-Xia-Xie-Xin-Tang	528/2,751 (19.2%)	0.80 (0.60–1.07)	0.80 (0.60–1.08)
Chuan-Qiong-Cha-Tiao-San	502/2,751 (18.3%)	0.74 (0.55–1.01)	0.78 (0.57–1.07)

Model 1: adjusted for age, gender, insured amount, and urbanization level.

Model 2: adjusted for age, gender, insured amount, urbanization level, comorbidities, and baseline drugs.

HR, hazard ratio; CHM, Chinese herbal medicine; CI, confidence interval.

*p-value < 0.05.

### Sensitivity Test

To verify the robustness of our results, serial sensitivity tests were performed. Supplementary Material S1 shows the sensitivity test results for different intervals between the index date and the initial diagnosis date of CVD. If we excluded the intervals of less than 30 days or 90 days, the HR of CVD was still significantly lower among CHM users. Supplementary Material S2 shows the sensitivity test for different intervals between the SLE diagnosis date and the index date. On excluding the intervals greater than 1 year, 3 years, or 6 years, the HR of CVD was still significantly lower among CHM user, with an especially lower HR of CVD in the interval less than 1 year (adjusted HR: 0.81; 95% CI 0.70–0.95; *p* = 0.009). Finally, if we restricted the cumulative days of CHM use to more than 30 days, the HR of CVD showed a significant further decrease in the CHM group (adjusted HR: 0.62; 95% CI 0.52–0.73; *p* < 0.001, Supplementary Material S3).

## Discussion

This nationwide population-based retrospective cohort study aimed to determine the effect of integrative therapy with CHM on the risk of CVD among patients with SLE in Taiwan. We finally included 5,502 patients initially diagnosed with SLE between 2004 and 2013 and followed up until December 2015. Multivariate analysis revealed a significant decrease in the risk of CVD among SLE patients using CHMs (adjusted HR: 0.83; 95% CI 0.73–0.95; *p* = 0.008). Thus, the study presented an evidence-based association between CHM use and reduced CVD risk among patients with SLE. Multivariate analysis of different types of CVDs also revealed a significant decrease in the risk of ischemic stroke in the CHM group (adjusted HR: 0.74; 95% CI 0.57–0.97; *p* = 0.032). However, there was no significant decrease in the risk of CAD (adjusted HR: 0.82; 95% CI 0.67–1.01; *p* = 0.060) or PAD (adjusted HR: 0.85; 95% CI 0.70–1.03; *p* = 0.097) in the CHM group after adjustment.

Chang et al. (2016) reported that the protective mechanism of a Chinese medicine polyherbal formula against endothelial injury among SLE patients may include suppression of endothelial injury biomarkers, vascular endothelial growth factor, and IL-18. In comparison with the basic molecular study, our population-based cohort study also showed a correlation between CHM use and protective effects against CVD among SLE patients. Many previous studies have evaluated the protective mechanism of CHMs against CVD. Berberine (the major ingredient of Coptis chinensis Franch.) can reduce blood glucose and lipid levels and may show anti-obesity effects (Xie et al., 2011). Berberine also has an antidiabetic effect (Zhang et al., 2015). Curcumin (the major ingredient of *Curcuma longa* L.) can modulate chronic inflammatory diseases (Wang et al., 2017). In addition, resveratrol (a natural compound extracted from *Reynoutria japonica Houtt*.) exerts antioxidant, anti-inflammatory, anti-obesity, and cardioprotective effects (Lyu et al., 2017). In one study, resveratrol demonstrated a potential atheroprotective effect by normalizing cholesterol efflux in macrophages exposed to plasma from SLE patients (Voloshyna et al., 2016).

The sensitivity test results revealed the robustness of our study. Under consideration of the restrictions of intervals between the index date and the CVD diagnosis date or between the SLE diagnosis date and the index date, the CHM group still showed significant reductions in the CVD risk. In addition, cumulative CHM use for more than 30 days resulted in a 38% decrease in CVD risk. In comparison with the 17% reduction in CVD risk in the CHM group without restriction of cumulative days, it seemed that a longer cumulative period of CHM use was correlated with a greater cardiovascular protective effect.

Among the top 10 frequently used single herbs and the top 10 frequently used polyherbal formulas, only Shu-Jing-Huo-Xue-Tang was associated with a significant protective effect against CVD in adjusted model 2. Shu-Jing-Huo-Xue-Tang is a formula of 17 single herbs, including *Glycyrrhiza glabra* L. (Gan Cao), *Angelica sinensis* (Oliv.) Diels (Dang Gui), *Paeonia lactiflora* Pall. (Bai Shao), *Rehmannia glutinosa* (Gaertn.) DC. (Sheng Di Huang), *Atractylodes lancea* (Thunb.) DC. (Cang zhu), *Achyranthes bidentata* Blume (Niu Xi), Citrus × aurantium L. (Chen Pi), *Prunus persica* (L.) Batsch (Tao Ren), *Clematis chinensis Osbeck* (Wei Ling Xian), *Conioselinum anthriscoides* “Chuanxiong” (Chuan Xiong), *Stephania tetrandra* S.Moore (Fang Ji), *Hansenia weberbaueriana* (Fedde ex H.Wolff) Pimenov and Kljuykov (Qiang Huo), Saposhnikovia divaricata (Turcz. ex Ledeb.) Schischk. (Fang Feng), Angelica dahurica (Hoffm.) Benth. and Hook.f. ex Franch. and Sav. (Bai Zhi), Gentiana scabra Bunge (Long Dan), Smilax glabra Roxb. (Fu Ling), and Zingiber officinale Roscoe (Sheng Jiang). Shu-Jing-Huo-Xue-Tang could activate blood and dispel wind in Chinese medicine theory. Indications of Shu-Jing-Huo-Xue-Tang are lumbago, joint pain, intramuscular pain, or whole body pain (Taiwan Herbal Pharmacopeia 3rd Edition Committee, 2019). A previous animal study revealed that Shu-Jing-Huo-Xue-Tang enhanced the anticoagulant effect of warfarin, and there might be potential bleeding risk while using Shu-Jing-Huo-Xue-Tang with anticoagulants in clinical practice (Yang et al., 2013). Thus, the mechanism underlying the cardiovascular protective effect of Shu-Jing-Huo-Xue-Tang might be related to its anticoagulant effect, and further clinical or basic studies are needed to clarify this.

The main finding of our study was the significant cardiovascular protective effect observed in the CHM group; however, the 10 most frequently used single herbs and 9 most frequently used polyherbal formulas did not show significant cardiovascular protective effects. One possible explanation for this discrepancy may be related to the fact that CHM prescriptions in clinical practice commonly include 2-3 polyherbal formulas with 3-4 single herbs. Thus, several single herbs and polyherbal formulas may have synergistic effects on the cardiovascular system. In Chinese medicine theory, CHMs may treat SLE patients by dispelling phlegm, regulating Qi, activating blood flow, or clearing heat according to individual’s constitution, which is similar to the mechanism of anti-inflammatory drugs.

Generally, there is no cure for autoimmune diseases (Tavakolpour, 2017), and complications of autoimmune diseases including CVD are worthy of attention striking. Integrative therapy combining CHMs may provide more options for clinical doctors due to opportunities of reducing CVD. CHMs contain abundant ingredients among numerus natural plants. We believe there might be potential value for further studies to investigate the effects of ingredients of CHMs on CVD.

The CHM group in our study excluded patients who visited Chinese medicine outpatient departments for acupuncture alone without CHM prescription. However, an NHIRD study has previously showed the protective effect of acupuncture on coronary heart disease among rheumatoid arthritis patients (Wu et al., 2018). Since both SLE and rheumatoid arthritis are systemic autoimmune diseases, further studies on the effect of acupuncture on CVD among SLE patients might be conducted.

The present study had several strengths. First, the National Health Insurance (NHI) is an obligatory universal health insurance program in Taiwan that covers inpatients and outpatients of Western medicine, dental services, and Chinese Medicine. The NHI covers more than 99% of the entire Taiwanese population (https://www.nhi.gov.tw/English). Thus, this was a population-based large-scale study. Second, the study was long-term cohort study with follow-up assessments from 2004 to 2015. The longest follow-up duration was nearly 12 years. In addition, we used the same immortal time for both the CHM and non-CHM groups by defining the index date for the comparable non-CHM group to avoid immortal time bias.

This study also had some limitations. First, approximately 5% of Chinese medicine clinics were not covered by the NHI (Ma et al., 2016). Thus, CHM use among patients with SLE may have been underestimated in this study. Second, due to limitations of the NHIRD study, the confounding factors for adjustment in multivariate analysis did not include health awareness, smoking history, or family history of CVD. Third, the difference in SLE disease activity between the two groups could not be completely eliminated in this observational study. Instead, we analyzed baseline drugs used 1 year prior to the index date, including the DMARDs (hydroxychloroquine, sulfasalazine, azathioprine, methotrexate, leflunomide, mycophenolic acid, cyclosporine, and cyclophosphamide). The adjustment of DMARD usage status in the multivariate analysis might partially reflect SLE disease activity.

## Conclusion

This study demonstrated an association between reduced CVD risk and integrative therapy combining CHMs with conventional treatment among SLE patients. Among the frequently used single herbs and polyherbal formulas, we found that Shu-Jing-Huo-Xue-Tang had a significant protective effect against CVD. Further randomized clinical trials might be necessary to confirm a definite causal relationship.

## Data Availability

The data analyzed in this study is subject to the following licenses/restrictions: The datasets used and analyzed during the current study are not publicity available, but are available from the corresponding author on reasonable request with the permission of the Ministry of Health and Welfare Data Science Center, Taiwan. Requests to access these datasets should be directed to https://dep.mohw.gov.tw/dos/np-2497-113.html.
