# Supplementary material for: Integrative Therapy Combining Chinese Herbal Medicines With Conventional Treatment Reduces the Risk of Cardiovascular Disease Among Patients With Systemic Lupus Erythematosus: A Retrospective Population-Based Cohort Study
Source: Front Pharmacol. 2021 Sep 27;12:737105. doi: 10.3389/fphar.2021.737105 (PMC8502802; doi:10.3389/fphar.2021.737105)
Supplement: Supplementary file 1 [file DataSheet1.docx]

**Integrative Therapy Combining Chinese Herbal Medicines with Conventional Treatment Reduces the Risk of Cardiovascular Disease among Patients with Systemic Lupus Erythematosus: A Retrospective Population-Based Cohort Study**

Han-Hua Yu^1,2^, Chia-Jung Hsieh^3^

^1^Division of Rheumatology, Allergy and Immunology, Linkou Chang Gung Memorial Hospital, Taoyuan, Taiwan

^2^Department of Chinese Medicine, Hualien Tzu Chi Hospital, Buddhist Tzu Chi Medical Foundation, Hualien, Taiwan

^3^Department of Public Health, Tzu Chi University, Hualien, Taiwan

Supplementary Material 1. Sensitivity test for the hazard ratios of cardiovascular disease of the different interval between the index date and the initial diagnosis date of cardiovascular disease.

|  | Patients | Patients with CVD | Crude HR  (95% CI) | Adjusted HR (95% CI) | |
| --- | --- | --- | --- | --- | --- |
|  |  |  |  | Model 1 | Model 2 |
| *Interval < 30 days excluded* | | | | | |
| Non-CHM group | 2728 | 446 | 1.0 | 1.0 | 1.0 |
| CHM group | 2734 | 390 | 0.82 (0.71-0.93)* | 0.82 (0.71-0.94)* | 0.83 (0.73-0.96)* |
| *Interval < 90 days excluded* | | | | | |
| Non-CHM group | 2692 | 410 | 1.0 | 1.0 | 1.0 |
| CHM group | 2710 | 366 | 0.83 (0.72-0.95)* | 0.83 (0.72-0.96)* | 0.85 (0.74-0.98)* |

Model 1: adjusted for age, gender, insured amount, and urbanization level.

Model 2: adjusted for age, gender, insured amount, urbanization level, comorbidities, and baseline drugs.

CVD, cardiovascular disease; HR, hazard ratio; CI: confidence interval; CHM, Chinese herbal medicine.

* p-value < 0.05.

Supplementary Material 2. Sensitivity test for the hazard ratios of cardiovascular disease of the different intervals between the systemic lupus erythematosus diagnosis date and the index date.

|  | Patients | Patients with CVD | Crude HR  (95% CI) | Adjusted HR (95% CI) | |
| --- | --- | --- | --- | --- | --- |
|  |  |  |  | Model 1 | Model 2 |
| *Interval > 1 year excluded* | | | | | |
| Non-CHM group | 1715 | 361 | 1.0 | 1.0 | 1.0 |
| CHM group | 1715 | 305 | 0.78 (0.67-0.91)* | 0.78 (0.67-0.91)* | 0.81 (0.70-0.95)* |
| *Interval > 3 years excluded* | | | | | |
| Non-CHM group | 2445 | 447 | 1.0 | 1.0 | 1.0 |
| CHM group | 2445 | 389 | 0.81 (0.71-0.93)* | 0.82 (0.71-0.94)* | 0.84 (0.73-0.96)* |
| *Interval > 6 years excluded* | | | | | |
| Non-CHM group | 2689 | 466 | 1.0 | 1.0 | 1.0 |
| CHM group | 2689 | 403 | 0.81 (0.71-0.92)* | 0.81 (0.71-0.93)* | 0.83 (0.73-0.95)* |

Model 1: adjusted for age, gender, insured amount, and urbanization level.

Model 2: adjusted for age, gender, insured amount, urbanization level, comorbidities, and baseline drugs.

CVD, cardiovascular disease; HR, hazard ratio; CI: confidence interval; CHM, Chinese herbal medicine.

* p-value < 0.05.

Supplementary Material 3. Sensitivity test for the hazard ratios of cardiovascular disease of the cumulative days of Chinese herbal medicine use more than 30 days.

|  | Pateints | Patients with CVD | Crude HR  (95% CI) | Adjusted HR (95% CI) | |
| --- | --- | --- | --- | --- | --- |
|  |  |  |  | Model 1 | Model 2 |
| Non-CHM group | 1820 | 341 | 1.0 | 1.0 | 1.0 |
| CHM group | 1820 | 230 | 0.60 (0.50-0.71)* | 0.60 (0.51-0.71)* | 0.62 (0.52-0.73)* |

Model 1: adjusted for age, gender, insured amount, and urbanization level.

Model 2: adjusted for age, gender, insured amount, urbanization level, comorbidities, and baseline drugs.

CVD, cardiovascular disease; HR, hazard ratio; CI: confidence interval; CHM, Chinese herbal medicine.

* p-value < 0.05.
